# Scientific meetings within a Benin Teaching Hospital: what type of research is being done? A pilot initiative to identify needs of health services research in a resource-limited country

**DOI:** 10.1186/1472-6963-14-S2-P150

**Published:** 2014-07-07

**Authors:** Alain Azondekon, Tanguy Bognon, Rafiou Lawani, Rodolph Vignon, Marc Fayomi, Albert Gnangnon, Benson Amaechi

**Affiliations:** 1 Military Teaching Hospital, Cotonou, Benin; 2 College of Medicine, Houdegbe North American University, Cotonou, Benin; 3 Benin Armed Forces Health Services, Cotonou, Benin

## Background

Research in health services is of huge concern especially in developing settings as economic issues and human resources management are critical in the context of the global financial crisis. The aim of this study is to evaluate the types of research done in a Military Teaching Hospital and hence identify the need for health services research in a resource-constrained environment.

## Material and methods

All presentations made during scientific meetings from June 2011 to December 2013 were reviewed and their domains of study (research) were identified. Scientific meetings at a Military Teaching Hospital were organized, in order to promote research within Benin Armed Forces Health Services, through monthly meetings. Semester and annual planning was done for presentations as each department was asked to propose 2 research conclusions per year. Research conclusions were programmed and presented as well as their perspectives.

## Results

During this period (30 months), 31 sessions were organized and 61 presentations were done. Speakers were from clinical, surgical, public health and administrative departments (17 in total), and were physicians (56%), health administrators (3%), public health specialists (26%), and human and social sciences specialists (13%). The research domains were enhancing care practices (61%), collaborative research between departments (13%), health economics (16%), and human resources management (10%).

The perspectives identified were mainly researchers training in methodology, focusing health services research on health economics especially in cost-analysis, improving health services organization in order to increase service performance.

## Conclusions

This original initiative in Benin Health services has shown the features of research in health services especially in a referral hospital. As training in health services research methodology and health economics are objectives to be attained, future planning will take into account a better overview of Benin Armed Forces Health Services and serve as a gold standard for the National Health Services in Benin.

